# Oxygen-Deficient Zirconia (ZrO_2−x_): A New Material for Solar Light Absorption

**DOI:** 10.1038/srep27218

**Published:** 2016-06-06

**Authors:** Apurba Sinhamahapatra, Jong-Pil Jeon, Joonhee Kang, Byungchan Han, Jong-Sung Yu

**Affiliations:** 1 Department of Energy Systems Engineering, DGIST, Daegu, 42988, Republic of Korea; 2 Department of Chemical and Biomolecular Engineering, Yonsei University, Seoul, 03722, Republic of Korea

## Abstract

Here, we present oxygen-deficient black ZrO_2−x_ as a new material for sunlight absorption with a low band gap around ~1.5 eV, via a controlled magnesiothermic reduction in 5% H_2_/Ar from white ZrO_2_, a wide bandgap(~5 eV) semiconductor, usually not considered for solar light absorption. It shows for the first time a dramatic increase in solar light absorbance and significant activity for solar light-induced H_2_ production from methanol-water with excellent stability up to 30 days while white ZrO_2_ fails. Generation of large amounts of oxygen vacancies or surface defects clearly visualized by the HR-TEM and HR-SEM images is the main reason for the drastic alteration of the optical properties through the formation of new energy states near valence band and conduction band towards Fermi level in black ZrO_2−x_ as indicated by XPS and DFT calculations of black ZrO_2−x_. Current reduction method using Mg and H_2_ is mild, but highly efficient to produce solar light-assisted photocatalytically active black ZrO_2−x_.

The ever-growing worries about future energy resources for the mankind stimulate the present scientific research community to develop new materials for sunlight harvesting. In general, zirconia (ZrO_2_) is not considered for sunlight absorption as it is a semiconductor with a wide band gap of around 5 eV and absorbs only ultraviolet (UV) light although ZrO_2_-based materials have promising application in several fields like thermal coating, optical coating, sensor, catalysis, energy conversion and storage, and biomedical applications^1^,^2^,^3^,^4^,^5^,^6^,^7^,^8^,^9^,^10^,^11^,^12^,^13^.

Recently, black TiO_2−x_ materials were achieved by creating oxygen vacancies and/or defects at surface using different methods such as H_2_ treatment, chemical reduction, chemical oxidation, electrochemical reduction, and anodization-annealing^14^,^15^,^16^,^17^,^18^,^19^,^20^,^21^,^22^,^23^. Fascinatingly, they exhibited an extended absorption in visible (VIS) and infrared (IR) instead of only UV light that is usually absorbed by white TiO_2_. After its discovery, black TiO_2−x_ finds several applications, but more importantly, it shows improved photocatalytic activity for H_2_ generation and environmental pollutant removal in comparison to its white counterpart. Such drastic change of the properties in black TiO_2−x_ was mainly ascribed to the high amount of oxygen vacancy and/or surface defects and Ti^3+^, which introduce new energy bands near Fermi level. Consequently, the electronic band gap decreases from 3.2 (anatase) to ~1 eV^14^,^15^. The concept of introduction of defects was also used for other metal oxides to improve their optical properties^24^,^25^,^26^,^27^,^28^,^29^,^30^.

In this context, the fabrication of oxygen-deficient black ZrO_2−x_ will be interesting to study the change in properties concerning sunlight harvesting. However, it will be more challenging as the electronic band gap of ZrO_2_ is higher than TiO_2_. Not only that, the oxygen vacancy formation energy of ZrO_2_ (820–880 kJmol^−1^ at the surface and 860 kJmol^−1^ for bulk) is also higher than that of TiO_2_ (530–580 kJmol^−1^ at the surface and 670 kJmol^−1^ for bulk)^2^,^31^. Thus, the previous studies on reduced and/or oxygen deficient ZrO_2−x_ have employed more drastic conditions like high-temperature evacuation, high temperature and pressure H_2_ treatment and Ar^+^ bombardment^2^,^32^,^33^,^34^,^35^,^36^,^37^,^38^,^39^,^40^,^41^, and mainly investigated the resulting change in electronic properties, but no solar light harvesting and photocatalytic studies have been performed. There is no meaningful report on fabrication of black ZrO_2−x_ for solar light harvesting to the best of our knowledge, which inspires us to develop black ZrO_2−x_ to perceive the changes of properties and photocatalytic activity.

## Results and Discussion

**Figure 1:**
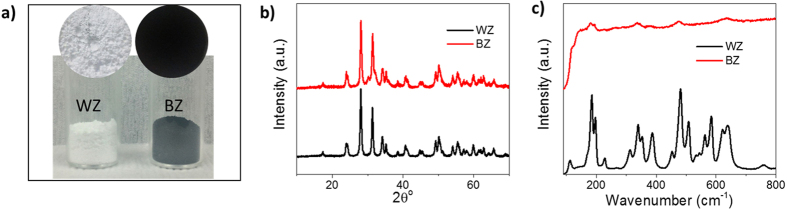
(**a**) Photograph of the powder samples indicating the color, (**b**) XRD patterns (**c**) Raman spectra of the WZ and BZ.

To study the possibilities of synthesis of black ZrO_2−x_ (BZ), we employed simple and mild two-step method that involves magnesiothermic reduction of commercially available monoclinic white ZrO_2_ (WZ) in the presence of 5% H_2_/Ar followed by HCl treatment to remove all the Mg species (see methods)^21^. The color of resulting zirconia material was black and named as BZ (Fig. 1a). The molar ratio of ZrO_2_ and Mg was optimized to get black ZrO_2−x_ and a trend of color change was observed (Supplementary Figure S1). Unless mentioned with a specific molar ratio of Mg, BZ was prepared with 1:1 molar ratio of ZrO_2_ and Mg and analyzed to examine the properties. The materials were first characterized using powder X-ray diffraction (XRD). The BZ shows almost pure monoclinic ZrO_2_ phase as like as the pristine white ZrO_2_ (Fig. 1b). The XRD pattern does not indicate any significant change in the crystal structure during magnesiothermic reduction. The crystallite size (Supplementary Table S1) of the BZ (19.6 nm) is also almost similar to that of WZ (20.2 nm). However, the drastic color change indicates some major alteration, possibly at the surface of the ZrO_2_ particles. This can be presumed as, during the reduction process, Mg was converted to MgO (Supplementary Figure S2) by taking up the surface oxygen, and thus, the chemical structure of ZrO_2_ can be disturbed at the surface. This indicates major alteration at the surface of BZ nanoparticles (NPs) in the form of defects.

**Figure 2:**
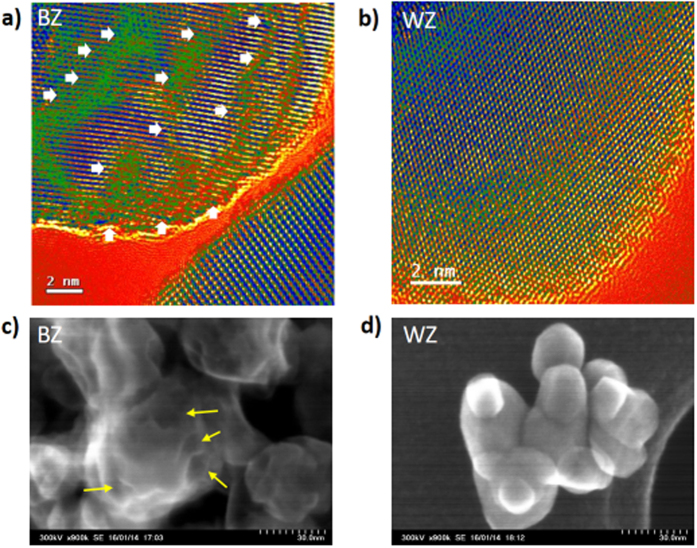
(**a,b**) High-resolution TEM images (The green colored areas indicated by white arrows show the surface defects.) and (**c,d**) high-resolution SEM images (the surface alteration indicated by arrow marks) of the BZ and WZ.

To study the surface alteration, the samples were further characterized by Raman spectroscopy as it is locally sensitive to the disorder in the first few atomic shells at the surface and lattice defects, which are not detectable in XRD analysis. Interestingly, the Raman spectra (Fig. 1c) of BZ are remarkably changed compared with that of WZ, and the corresponding peaks are broadened, and peak intensity is enormously decreased. This result supports the presumption about surface alteration during reduction, which results in black coloration and also indicates the possible formation of huge oxygen vacancy at the surface. The phenomena can be understood as the crystal arrangements of the surface Zr-O units are disturbed during reduction due to oxygen vacancy, which results in the disordered surface. Further, high resolution-transmission electron microscopy (HR-TEM) images of BZ (Fig. 2a and Supplementary Figure S3 d–I) clearly demonstrate the formation of random defects on BZ NPs unlike WZ, which shows well-defined crystalline lattice structure (see Fig. 2b and Supplementary Figure S3 a–c). Furthermore, the line profile (Supplementary Figure S4) of the HR-TEM images of BZ clearly visualizes the presence of disorder lattice pattern in the defected area. The size of the WZ and BZ NPs was almost identical with around 25–30 nm. The surface alteration was further witnessed by high resolution-scanning electron microscopy (HR-SEM) images (see Fig. 2c,d), which illustrate that the smooth and clean surface of WZ NPs changed to uneven and hollow surface in BZ as indicated by arrow marks. These HR-TEM and HR-SEM images may be the first visualized evidence of surface defects in BZ. The distribution, size and shape of the defects or disorders are clearly visualized by these techniques.

**Figure 3:**
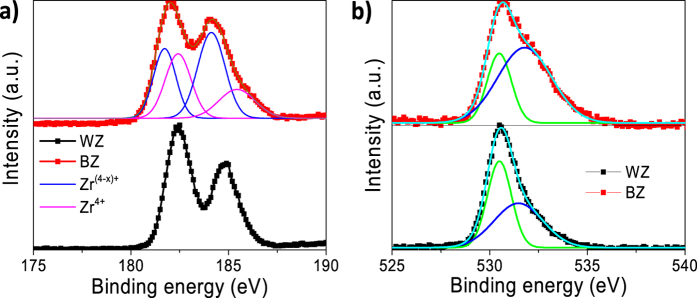
High-resolution (**a**) Zr 3d and (**b**) O 1s XPS spectra of WZ and BZ.

Furthermore, X-ray photoelectron spectroscopy (XPS) was also investigated to study the alteration of the bonding environment of Zr and O at the surface. The high resolution XPS spectrum of Zr 3d electron (Fig. 3a) of BZ shows two peaks at 182.0 and 184.2 eV in accordance with the counterpart peaks (182.4 and 184.8 eV) of WZ corresponding to 3d_5/2_ and 3d_3/2_ electron of Zr^4+^, respectively^42^. Further analysis of deconvoluted BZ’s Zr 3d XPS spectrum can illustrate the presence of another set of peaks corresponding to reduced Zr species, i.e. Zr^(4−x)+^. However, no indication was observed for metallic Zr (~178 eV)^42^. Interestingly, the high-resolution O 1 s XPS spectrum (Fig. 3b) of the BZ shows a long tail towards higher binding energy compared with the white sample. On deconvolution of the spectrum, it resolved into two distinguishable peaks centered at 530.5 and 531.9 eV corresponding to the lattice oxygen and non-lattice oxygen (*i.e.* oxygen vacancy), respectively. It can be also observed that the amount of the oxygen vacancy is higher in BZ than in WZ. The relative amount of the oxygen vacancy is 71% at the surface of BZ while 43% for WZ as obtained from the corresponding peak area (Supplementary Table S2). Therefore, these results clearly indicate the formation of large amounts of oxygen vacancy during the synthesis of black ZrO_2−x_ and well agree with the XRD, Raman and HR-TEM analysis.

Formation of the huge amount of oxygen vacancy and reduced Zr species is further evidenced by electron paramagnetic resonance (EPR) analysis (Supplementary Figure S5). The EPR spectrum of BZ shows a very sharp signal at g = 2.00 and a small peak at g = 1.98 corresponding to free electron coming from oxygen vacancy and Zr^3+^, respectively, whereas WZ does not show any significant EPR signal^35^,^43^. The presence of oxygen deficiency in BZ was further evidenced by thermogravimetric analysis (Supplementary Figure S6) in the presence of oxygen, which shows a weight increment after ~150 °C, indicating the oxygen uptake. The full XPS survey spectra of the BZ and WZ (Supplementary Figure S7) displayed no peak for Mg, further confirming the absence of Mg in the BZ. In addition, N_2_ adsorption-desorption isotherms also indicated no significant alteration (Supplementary Figure S8 and Table S3) in textural properties of BZ compared with WZ. The BET surface areas are determined to be 24 and 25 m^2^g^−1^ for WZ and BZ, respectively, indicating little change in physical surface properties between WZ and BZ.

**Figure 4:**
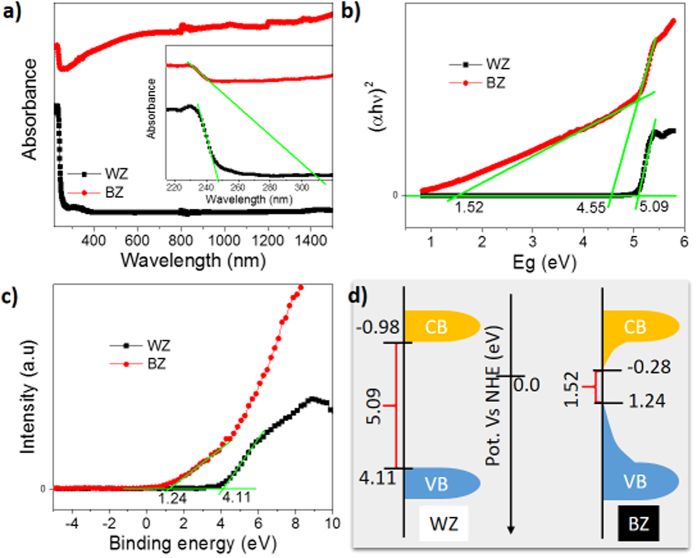
(**a**) UV-VIS DRS spectra and enlarged UV-VIS DRS spectra (inset), (**b**) Tauc plots obtained from the UV-VIS data (see supplementary information for detail), (**c**) valence band (VB) XPS spectra, and (**d**) the probable band energy diagrams of black (BZ) and white (WZ) zirconia samples. The approximate positions of VB top and conduction band (CB) bottom are calculated from the VB XPS and band gap values (see supplementary Table S4).

The UV-VIS-IR absorption spectra of as-prepared BZ samples were measured to study the absorbance of as-synthesized black zirconia. As expected, the black ZrO_2−x_ sample exhibited a drastic enhancement (Fig. 4a) in the light absorption in the range of VIS and IR region compared with its white counterpart which absorbs only UV light and shows a sharp absorption peak at ~246 nm. The increment in light absorption also increases with the Mg amount used during sample preparation in accordance with the color change trend of white to gray to black (Supplementary Figure S1 and S9). The band gap of the samples was determined from Tauc plot (Fig. 4b) obtained from the UV-VIS data. The band gap of the BZ is 4.55 eV, and an adjusted band gap is obtained to be around 1.52 eV, which may correspond to the black color of the sample, while the band gap of WZ is 5.09 eV. Furthermore, the position of the valence band (VB) top was determined by VB XPS spectra (Fig. 4c). It clearly indicates the movement of the VB top to upwards and also the extended tail formation to decrease the band gap of BZ compared with WZ. The approximate positions of the VB top and CB (conduction band) bottom were calculated (Supplementary Table S4) from the above results, and the probable band energy diagram is portrayed in Fig. 4d. This drastic change in band gap due to formation of new energy states near VB top and CB bottom can be attributed to the presence of high oxygen vacancy and Zr^3+^ in BZ^16^,^34^,^44^.

**Figure 5:**
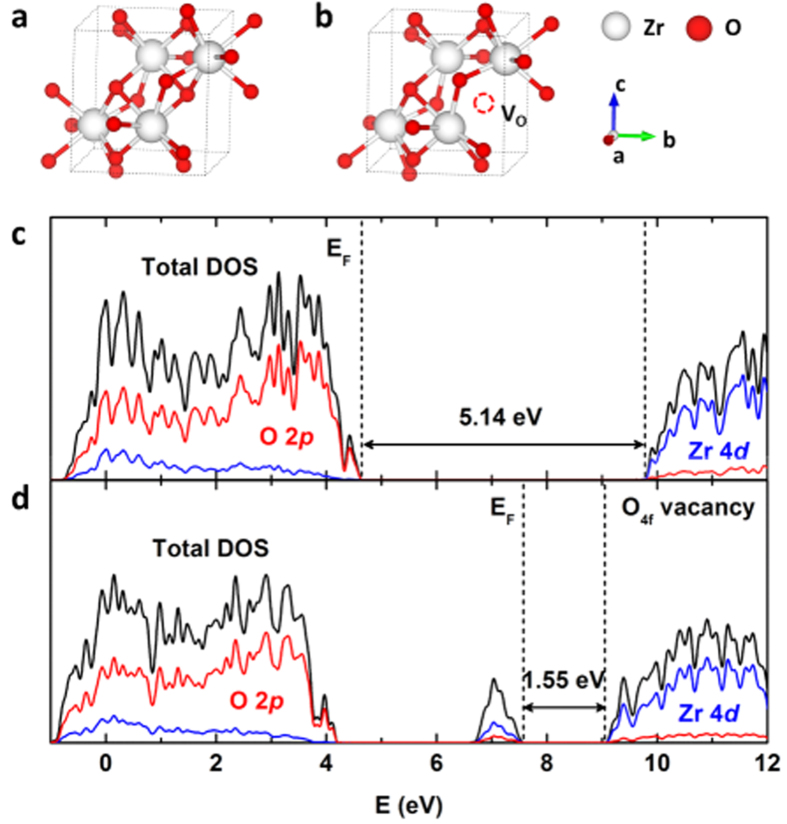
(**a,b**) Monoclinic ZrO_2_ crystal structures with and without oxygen vacancy, respectively. (**c,d**) The DOS of ZrO_2_ with no oxygen vacancy and O4f vacancy, respectively. Black, blue, and red lines represent total DOS, Zr 4d PDOS, and O 2p PDOS, respectively. The left and right dashed lines represent the Fermi level and conduction band minimum, respectively.

The experimental results were further confirmed by density functional theory (DFT) calculation. Figure 5a shows a monoclinic ZrO_2_ crystal structure (WZ model) composed of 12 atoms per unit cell. The monoclinic phase has two kinds of non-equivalent oxygen atoms: 4-fold coordinated (O_4f_) and 3-fold coordinated (O_3f_)^2^,^31^,^44^. One O_4f_ vacancy was used for a BZ model (Fig. 5b) since the oxygen vacancy formation energy was calculated to be 0.1 eV lower than that of the O_3f_. The band gap of a WZ was calculated as 5.14 eV by HSE06 method as shown in Fig. 5c, which is in excellent agreement with our experimental measurement of 5.09 eV. The results indicate that the upper VB largely consists of O 2*p* states while the lower part of the CB is mainly from Zr 4*d* states. The electronic structures of the BZ model systems were illustrated in Fig. 5d. It clearly depicts that new density of states (DOS) appears at the CB bottom and VB top, respectively. Band gap of BZ was evaluated as 1.55 eV, consistent with experimentally measured 1.52 eV. The projected density of states (PDOS) of 4*d* band of Zr implies that Zr^3+^ created by oxygen vacancies mainly contributes to the CB tail, as similarly for the CB tail of a black TiO_2−x_^45^. The VB mid-gap states are hybridized from both O 2*p* orbitals and Zr 4*d* orbitals as the result of disordered states of black ZrO_2−x_^31^, like black TiO_2−x_^15^,^16^,^46^,^47^. The above results confirm that the disordered states of ZrO_2−x_ were developed during magnesiothermic reduction process, which creates large amount surface defects (as nicely visualized in HR-TEM and HR-SEM images) via oxygen vacancies.

These new DOS at VB and CB tails can effectively block the recombination of electrons and holes and enable absorption of sunlight over a wider range of wavelengths including visible-light. Photoluminescence (PL) spectra (Supplementary Figure S10) were recorded using the same excitation wavelength (325 nm) and same slit (5 nm). The intensity of the PL spectrum corresponding to the black sample is much less than that of the white sample, although the peak positions are almost the same, which indicates the slower rate of electron-hole recombination, which can be ascribed to the result of electron and hole trapped in the newly formed energy bands at the CV bottom and VB top, respectively^44^,^48^.

The enhanced solar light absorption of the BZ was further characterized by photocatalytic degradation of Rhodamine B (RhB) in the presence of simulated solar light (1 sun, AM 1.5G). The results (Supplementary Figure S11) clearly indicate that WZ has almost no degradation ability in the presence of solar light, whereas the BZ shows sufficient degradation of RhB, which can be attributed to the improved solar light absorbance of BZ compare with WZ.

**Figure 6:**
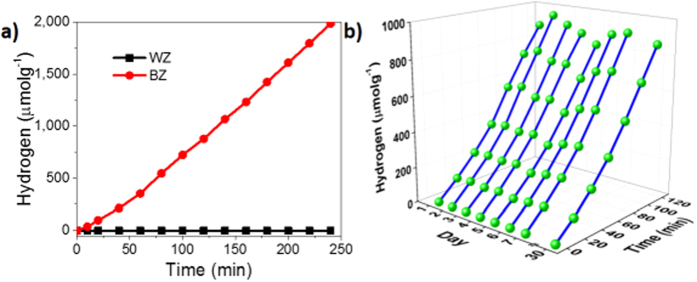
(**a**) Continuous generation of H_2_ from methanol-water solution by WZ and BZ and (**b**) stability study under simulated solar light (1 sun, AM 1.5 G).

The BZ was also investigated for the H_2_ generation from water using photo-deposited Pt NPs (~1.0 wt%) as a co-catalyst and methanol as a sacrificial reagent under simulated sunlight (1 sun, AM 1.5G). The BZ generated significant amount of H_2_ with a rate of 505 μmolg^−1^h^−1^ while no H_2_ was obtained from WZ (Fig. 6a). The calculated solar to hydrogen (STH) efficiency is 0.11% with respect to 1 sun incident power and present reaction setup (see Supplementary for detail)^49^. The H_2_ generation ability of BZ in sunlight is directly accredited to the drastically improved optical properties (solar light absorbance) and low band gap. Formation of the new DOS in the energy level enables the BZ to absorb the visible light and also generate H_2_ by water splitting under solar light. The stability test (Fig. 6b) for BZ demonstrated that almost similar amount of H_2_ produced in each run (120 min), which indicates the excellent stability (up to 30 days) of the black zirconia samples. Here, it should be mentioned that the color, absorbance, and H_2_ generation ability were almost the same after 1 month when samples were stored in the normal atmosphere in sample vial without any extra precaution.

The H_2_ production ability was further investigated for the samples prepared with the different ratios of Mg with respect to ZrO_2_ and in different conditions (Supplementary Figure S12). The results suggested that the ratio (1:1) can be considered as an optimum ratio. Further, we have synthesized reduced zirconia using only hydrogen (5% H_2_/Ar), only Mg (in Ar), and only Ar in the same reaction conditions to study the uniqueness of the present methods (see methods for detail).

All the materials were studied for the hydrogen production using the same experimental conditions. Interestingly, the material prepared using only Ar did not show any hydrogen production, whereas the materials prepared by only Mg and hydrogen show a little amount of hydrogen in the same experimental conditions under the solar light. These results clearly demonstrate the requirement of both Mg and H_2_ for the preparation of photocatalytically active black ZrO_2−x_. These results also indicate that controlled hydrogen reduction may also achieve good ZrO_2−x_ photocatalyst despite low reducibility with H_2_ mentioned in the previous study^2^. It also makes us more curious to know why Mg and H_2_ both are required and how they function. One may suggest that H_2_ is absorbed on the defect site created by Mg and form Zr-H active species for photocatalysis like Ti-H in hydrogenated black TiO_2−x_^14^,^50^. We are further working to find out the probable reason and understand the involved chemical reactions in our lab.

In conclusions, oxygen-deficient black zirconia (ZrO_2−x_) was prepared via the magnesiothermic reduction in H_2_/Ar atmosphere, which resulted in a drastic increment in solar light absorption and band gap decrement (to 1.52 from 5.09 eV for white ZrO_2_). A prominent surface alteration in the form of defects or disorders associated with the oxygen vacancy in black zirconia was well characterized and nicely visualized by high resolution microscopic techniques. Excitingly, the oxygen-deficient black ZrO_2−x_ shows good photocatalytic performance for RhB degradation and H_2_ production under simulated solar light (AM 1.5G) while white ZrO_2_ fails. Thus, white ZrO_2_, an inactive materials for sunlight harvesting has been transformed to an active black ZrO_2−x_. The employed process involves a simple and mild magnesiothermic reduction at 650 °C in presence of H_2_/Ar. The results indicate that both Mg and H_2_ are necessary for the preparation of efficient solar light-assisted photocatalytically active black ZrO_2−x_. The findings are highly important and innovative with respect to sunlight harvesting since this work presents the first report on solar light-assisted photocatalytic activity of black ZrO_2−x_ and can be considered as proof of concept for developing more novel materials for solar energy utilization. Our developed black ZrO_2−x_ material could be interesting for not only solar light-assisted photocatalytic application but also in other solar energy applications as well as applications like catalysis, sensors, energy conversion and storage, coating, and biomedical tests. This material offers a new choice, and the study will unveil a new direction to the scientific community working in diverse areas of material science and applications.

## Methods

### Preparation of oxygen-deficient black ZrO_2−x_

Commercially available monoclinic (as observed in XRD analysis) nano ZrO_2_ (WZ) was purchased from Sigma-Aldrich and used as it is. Well-mixed sample with a proper molar ratio of ZrO_2_ and magnesium powder was placed in a tube furnace and then heated with a ramp of 2.5 °C/min and maintained at 650 °C for 4 h in 5% H_2_/Ar atmosphere. After the annealing treatment, the sample was etched for 24 h in 2.0 M HCl solution. Then, the sample was washed with sufficient amount of water to remove the acid, and the removal of Cl^−^ ion was checked by AgNO_3_ solution. The washed solid mass was dried at 80 °C for 24 h to obtain black zirconia (BZ). The molar ratio of ZrO_2_ and Mg was varied while other experimental conditions was kept the same to get different black ZrO_2−x_ samples and a trend of color change was observed (Supplementary Figure S1). Unless mentioned with a specific molar ratio of Mg, the BZ prepared with 1:1 molar ratio of ZrO_2_ and Mg was analyzed to examine the properties. Furthermore, a set of different samples were prepared (i) in only Ar atmosphere in absence of any H_2_ and Mg, (ii) using 5% H_2_/Ar atmosphere in the absence of Mg, and (iii) in the presence of Mg (1 mole ratio with respect to ZrO_2_) in Ar atmosphere without H_2_ at 650 °C for 4 h.

### Characterization techniques

Powder X-ray diffraction data were recorded using a Rigaku Smartlab diffractometer with Cu-Kα (0.15406 nm) operated at 40 kV and 30 mA at a scan rate of 4° min^−1^. Raman analysis was carried out using a Raman spectrometer (NICOLET ALMECA XR), manufactured by Thermo Scientific. A 532 nm laser beam was used for excitation. High resolution-transmission electron microscopy (HR-TEM) images were collected using Hitachi HF−3300, operated at 300 kV. The same instrument was also used to obtain the high resolution-scanning electron microscopy (HR-SEM) images. The absorption spectra of the samples were recorded by using an ultraviolet-visible-near infrared (UV-VIS-NIR) spectrophotometer (CARY5000) manufactured by Agilent Technology. X-ray photoelectron spectroscopy (XPS) was performed using an ESCALAB 250 XPS System with a monochromated Al Kα (150 W) source. The energy scale is aligned by using the Fermi level of the XPS instrument (4.10 eV versus absolute vacuum value). The photoluminescence spectra were obtained from a Cary Eclipse Fluorescence Spectrophotometer (Agilent Technologies). The nitrogen adsorption–desorption isotherms of the samples were measured at −196 °C using a Micromeritics ASAP 2460 accelerated surface area and porosity analyzer after the samples were degassed at 150 °C to 20 mTorr for 12 h. The specific surface area was determined based on Brunauer–Emmett–Teller (BET) method from nitrogen adsorption data in the relative pressure range from 0.05 to 0.2. The electron paramagnetic resonance spectra were recorded on a Bruker EMX plus spectrometer in the X band CW electron paramagnetic resonance (EPR) spectroscopy using 0.94 mW microwave power with 9.646 GHz microwave frequency at −253 °C. Thermal Gravimetric Analysis (TGA) was carried out on a Bruker TG-DTA3000SA thermal analyzer at a heating rate of 10 °C/min under flowing O_2_, increasing from room temperature to 700 °C.

### Photocatalytic degradation of rhodamine B (RhB)

50 mg of zirconia photocatalyst was dispersed into 50 ml of 1 ppm RhB aqueous solution. The mixture solution was taken into a quartz reactor, and the quartz reactor was placed under solar light (1 Sun, 100 mWcm^−2^) simulated by a solar simulator (Newport, LCS-100) embedded with 100 W Xenon lamp and AM1.5G filter in continuous stirring condition. The sample was collected time to time and the solid was separated by centrifugation. The absorbance of the solution was analyzed in UV-VIS spectrophotometer. The concertation of the solution was calculated from the calibration plot of concentration vs absorbance obtained from solutions of known concentrations. The results may vary within ±5%.

### Photocatalytic Pt deposition

A certain amount of zirconia catalyst was taken into 50 ml 20% methanol-water solution in a closed gas circulation system. An appropriate amount of H_2_PtCl_6_. 6H_2_O was added. The UV light irradiation was obtained from a 450 W Xenon lamp (Newport) and used for the deposition of Pt over zirconia under Ar atmosphere. The freshly prepared Pt-deposited catalyst was collected, washed with water, dried at 80 °C and used for further study.

### Photocatalytic H_2_ generation

50 mg of ~1.0 wt% Pt-loaded zirconia photocatalyst was added to 20% methanol-water solution (50 ml,) in a closed gas circulation system. The reactor was placed under the solar light irradiation (1 Sun, 100 mWcm^−2^) obtained from a solar simulator (Newport, LCS 100) embedded with 100 W Xenon light source and AM1.5G filter. The approximate illuminated area was 15.75 cm^2^. Methanol was used as a sacrificial reagent to prevent the recombination by scavenging the hole and the surface back reaction by blocking oxygen production. The amount of H_2_ generated was determined by online gas chromatography (Bruker 450 GC) system using TCD detector connected to the reactor. The reaction was carried out at room temperature (25 °C) under Ar atmosphere. The stability of the catalyst system was studied under the identical reaction conditions as stated above. The solution was stored in the normal ambient condition in a closed container and studied for 2 h in the different days. Before each run, the volume of the solution (50 ml) was made up by adding extra methanol to the solution. The results may vary within ±5%.

### DFT Calculation

Density functional theory (DFT) calculations were performed with the Perdew-Burke-Ernzerhof exchange-correlation functionals^51^ and projector augmented wave^52^ generated pseudo-potentials, as implemented in the Vienna *ab-initio* simulation package (VASP)^53^. Plane-wave energy with cutoff energy of 500 eV was used to expand Kohn-sham orbitals^54^. A gamma-centered 6 × 6 × 6 *k*-point scheme was applied to integrate Brillouin zone and each calculation was continued until the forces acting on all atoms were less than 0.02 eV Å^−1^. To accurately evaluate the band gap of BZ, the screened hybrid functionals were used as proposed by Heyd-Scuseria-Ernzerhof (HSE06)^55^.

## Additional Information

**How to cite this article**: Sinhamahapatra, A. *et al*. Oxygen-Deficient Zirconia (ZrO_2−x_): A New Material for Solar Light Absorption. *Sci. Rep.* **6**, 27218; doi: 10.1038/srep27218 (2016).

## Supplementary Material

Supplementary Information
