# Supplementary material for: Oxygen-Deficient Zirconia (ZrO2−x): A New Material for Solar Light Absorption
Source: Sci Rep. 2016 Jun 6;6:27218. doi: 10.1038/srep27218 (PMC4893729; doi:10.1038/srep27218)
Supplement: Supplementary Information [file srep27218-s1.pdf]

Supplementary Information

Oxygen-Deficient Zirconia (ZrO_{2-x}): A New Material for Solar Light Absorption

Apurba Sinhamahapatra[1], Jong-Pil Jeon[1], Joonhee Kang[1], Byungchan Han[2,*] and Jong-Sung Yu[1,*]

[1] *Department of Energy Systems Engineering, DGIST, Daegu, 42988, Republic of Korea. E-mail: jsyu@dgist.ac.kr*

[2] *Department of Chemical and Biomolecular Engineering, Yonsei University, Seoul, 03722, Republic of Korea. E-mail: bchan@yonsei.ac.kr*

Color of the samples

Color	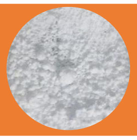	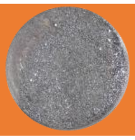	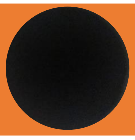	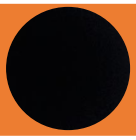
Molar ratio (ZrO_2: Mg)	1:0	1:0.5	1:1	1:1.3

Figure S1. Photographs of different powder samples obtained using different molar ratios of ZrO_2 and Mg, indicating the gradual color change from white to grey to black.

- **XRD analysis**

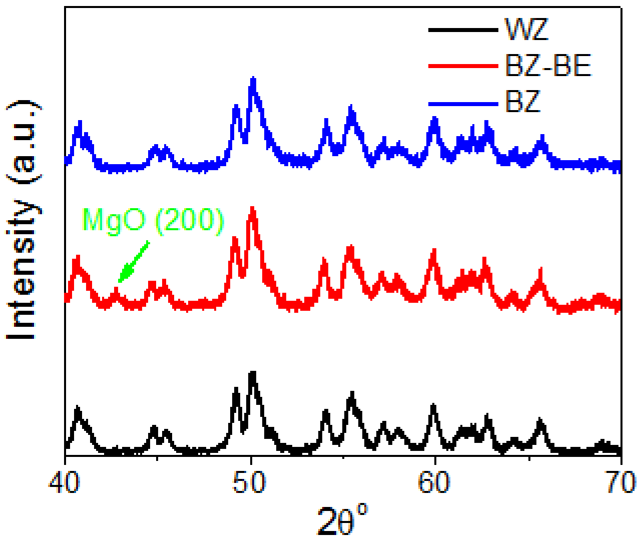

Figure S2. XRD patterns of as-synthesized samples before (BZ-BE) and after HCl etching (BZ) along with WZ, showing the formation of MgO from Mg in BZ-BE sample obtained before HCl etching. The results may suggest that Mg takes up the oxygen from ZrO_2 to form MgO and creates oxygen vacancy in resulting black ZrO_{2-x}.

Calculation of crystallite size

Table S1: Calculation of crystallite size

100 % peak in XRD	White ZrO_2 (WZ)	Black ZrO_{2-x} (BZ)
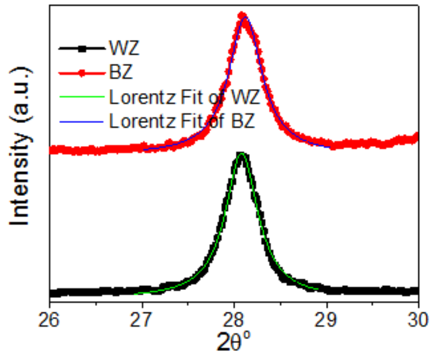	2θ = 28.08°<br>With= 0.423<br>*Dp* = 20.23 nm	2θ = 28.1°<br>With=0.436<br>*Dp* = 19.6 nm

We have calculated the crystallite size using the Scherrer equation from XRD data

$$D_p = \frac{0.94\lambda}{\beta_{1/2} \cos\theta}$$

where Dp = average crystallite size, $\beta_{1/2}$ = line broadening at half the maximum intensity, in radians, θ = Bragg angle, and λ = X-ray wavelength = 0.15406 nm

The BZ shows almost pure monoclinic ZrO_2 phase as like as the pristine white ZrO_2 (**Figure** 1b). The XRD pattern does not indicate any residual Mg species in the final materials, which also indicates the formation of pure black ZrO_{2-x} without any metal doping. No peak was also observed for sub-oxides of Zr. The similarity in XRD of the black and white zirconia indicates no significant change in the crystal structure during magnesiothermic reduction. The crystallite size (calculated using Scherer equation, see Table S1,) of the BZ (19.6 nm) is also almost similar to that of WZ (20.2 nm). However, the drastic color change indicates major alteration, possibly at the surface of the ZrO_2 particles. This can be presumed as during the reduction process, Mg is converted to MgO by taking up the surface oxygen, and thus, the chemical structure of ZrO_2 can be disturbed at the surface as observed in the case of black TiO_2. The formation of MgO can be evidenced by the presence of MgO peak in XRD pattern of the as-synthesized sample. This indicates major alteration at the surface of BZ nanoparticles (NPs) in the form of defects.

➢ HR-TEM analysis

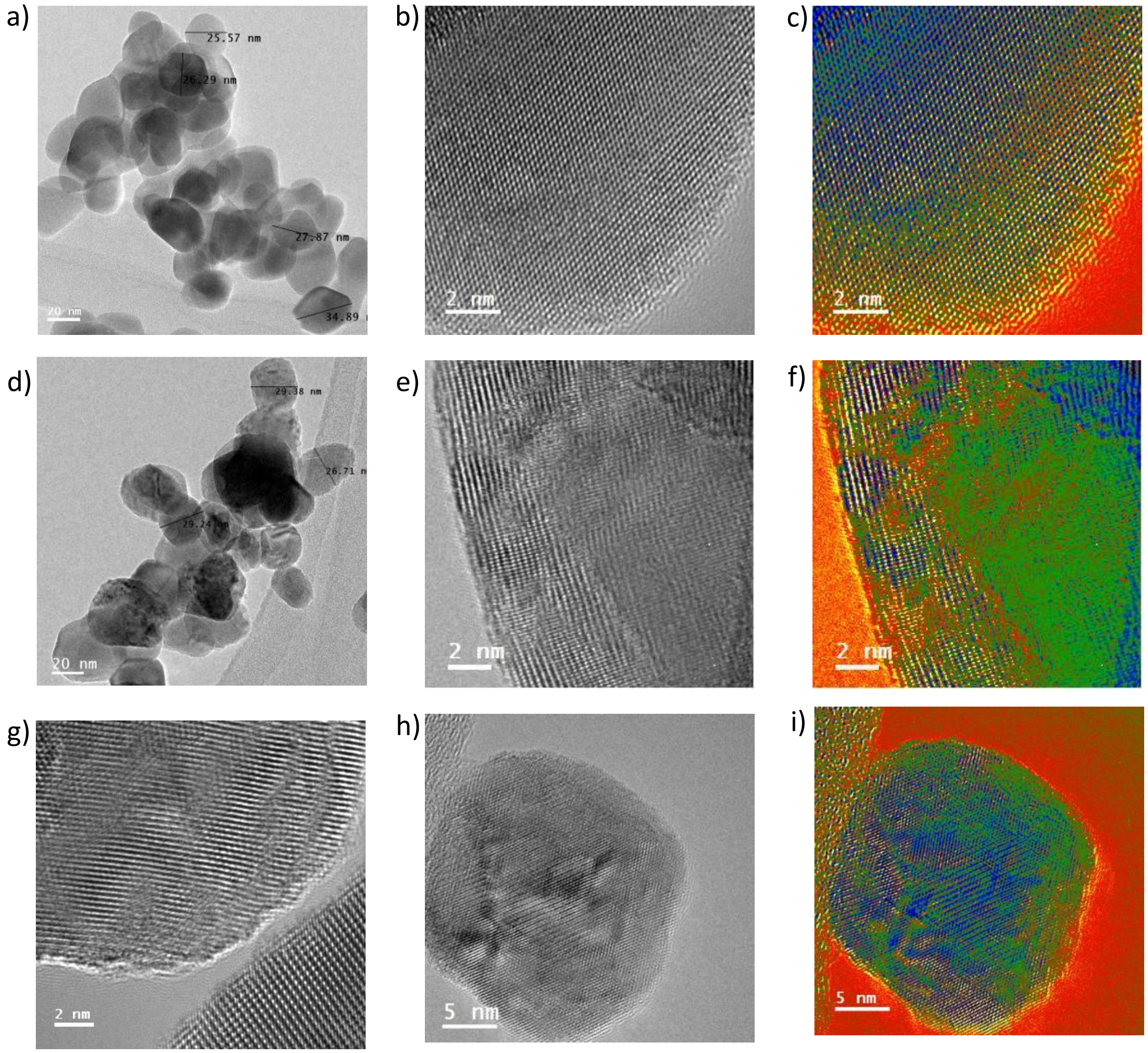

Figure S3. (a-c) HR-TEM images of WZ showing the ZrO_2 nanoparticles (25-35 nm) with a smooth surface and well define lattice structure. (d-i) HR-TEM images of BZ showing the presence of surface defects (green colored regions in the color images) unlike the WZ.

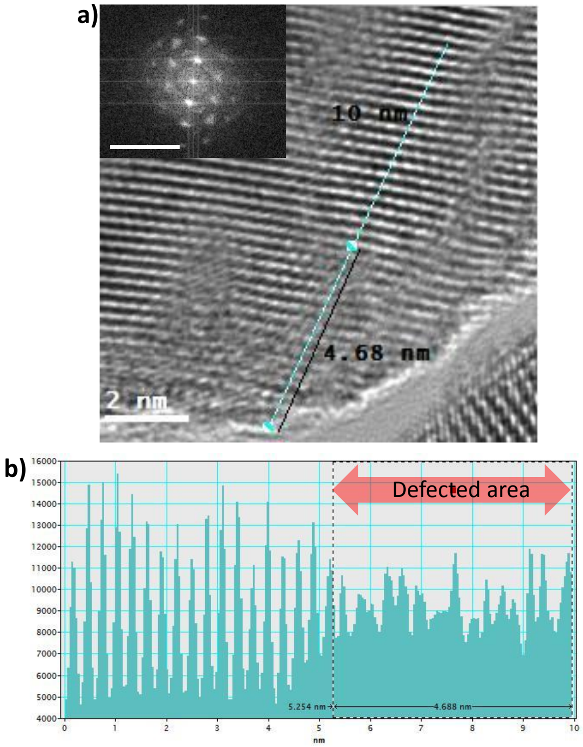

Figure S4. (a) HR-TEM image of BZ showing the disordered lattice structure and (b) the line profile of the marked (line) area in image a. The corresponding Fast Fourier Transform (FFT) is provided in inset (a).

➢ XPS, TGA and EPR analysis

Table S2. Calculation of oxygen vacancy from the XPS result

O 1s XPS spectrum	Sample	Relative peak area		% 'O' vacancy
		Lattice 'O'	'O' vacancy	
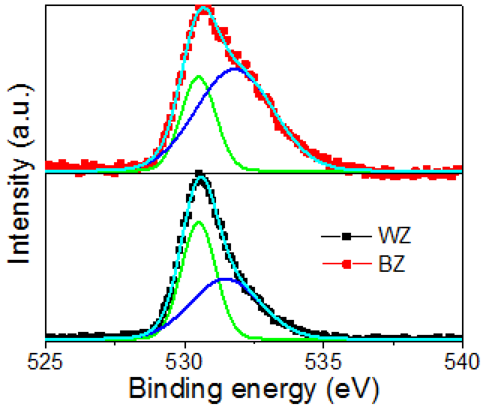	WZ	1.45	1.11	43
	BZ	0.87	2.09	71

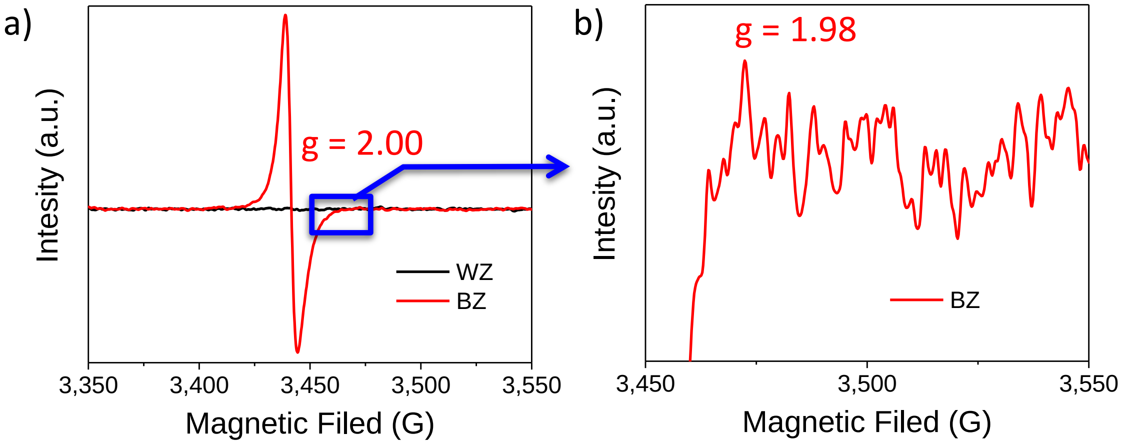

Figure S5. (a) EPR spectra of the white and black zirconia samples. (b) Enlarge EPR spectrum of BZ. The spectra were recorded at -253℃ using 0.94 mW microwave power and 9.646 GHz microwave frequency.

The g factor is calculated using the following equation:

$$hv = g\beta B \text{ or } g = \frac{hv}{\beta B} \text{ or } g = \frac{71.4484 \times v\ (GHz)}{B\ (mT)}$$

where β is the constant (Bohr magneton), h is the Planck constant, v is the microwave frequency, and B is the magnetic field.

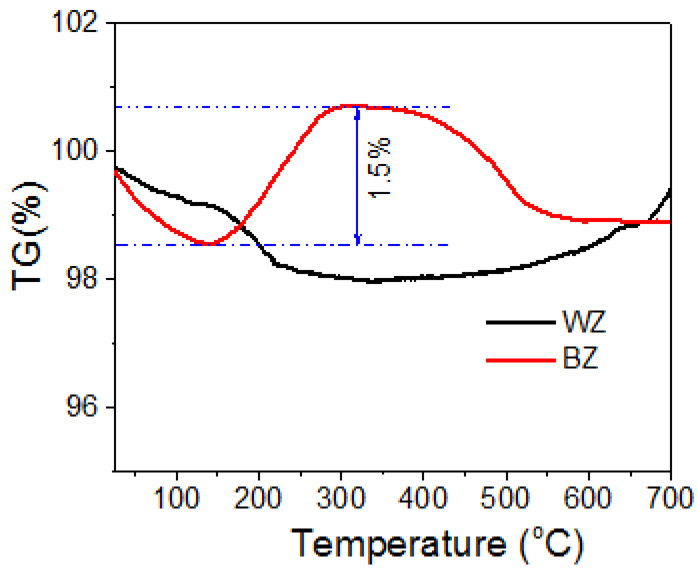

Figure S6. TGA profiles of the white and black zirconia samples recorded under oxygen atmosphere with 10 °C/min heating rate.

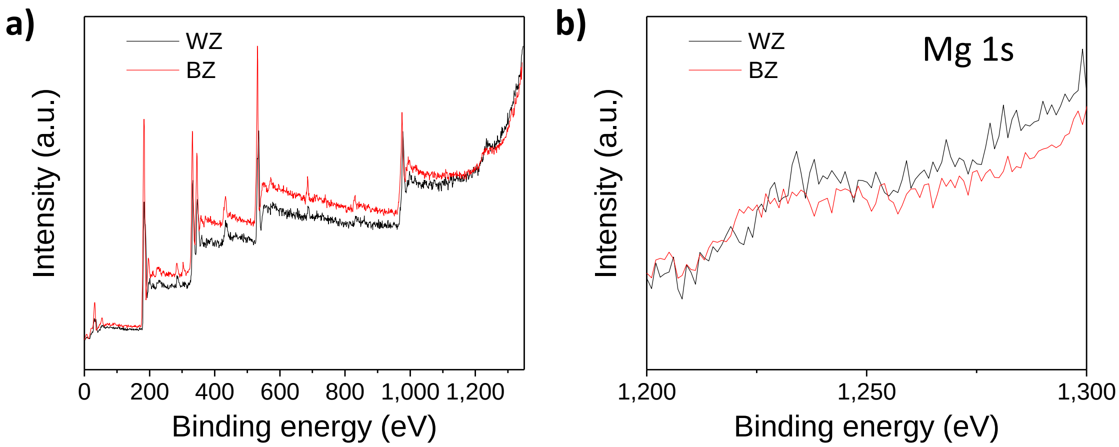

Figure S7. (a) Full XPS survey spectra and (b) Mg 1s scans of the white and black samples. This result clearly indicates the similarity of the elemental presence in the white and black zirconia and also the absence of Mg species.

➢ **Surface area analysis**

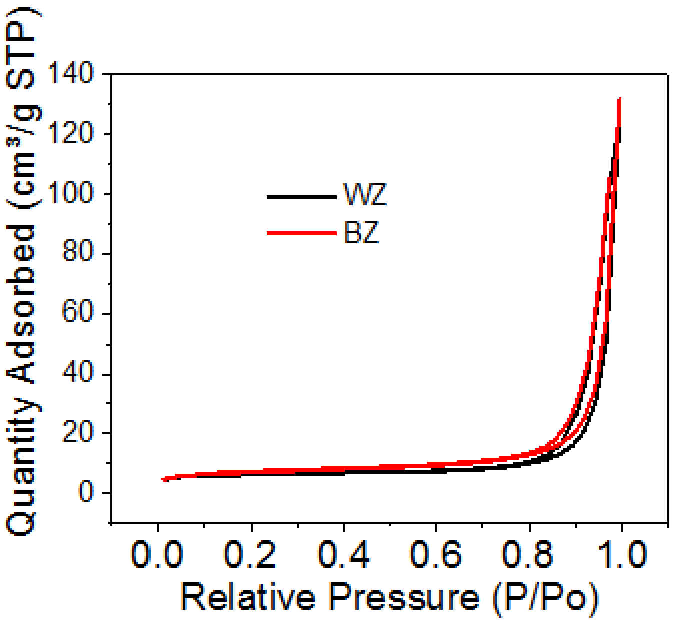

Figure S8. N_2 adsorption-desorption isotherms of the white and black zirconia samples.

Table S3. Results obtained from N_2 isotherm analysis

Parameters	WZ	BZ
BET surface area (m^2g^{-1})	24	25
BJH adsorption pore volume (cm^3g^{-1})	0.19	0.20
BJH adsorption average pore size (4V/A) (nm)	45.3	42.9

➢ **UV-VIS DRS and photoluminescence spectra**

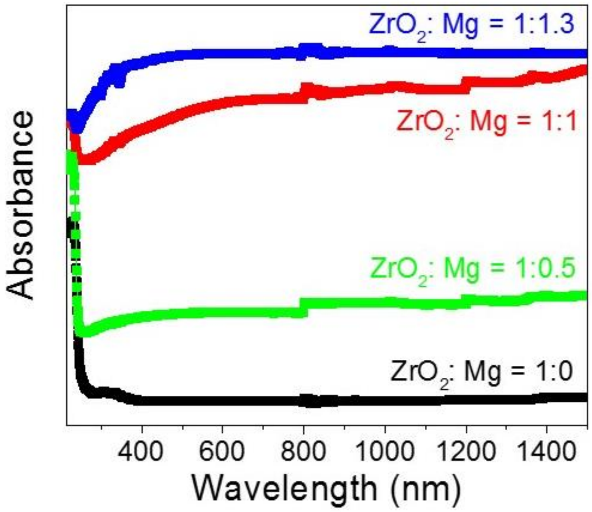

Figure S9. UV-VIS DRS spectra of different zirconia samples prepared using different Mg ratios, showing the continuous enhanced absorbance in VIS and IR region with the increase of Mg amount in accordance with color trend (see supplementary **Figure** 1)

Band gap calculation

The band gap of the samples was calculated using Tauc plot of $(\alpha h\nu)^2$ vs hν, where α is the absorption coefficient, h is Plank constant, and ν is the wavenumber. The value of hν is obtained by converting the wavelength (λ) value. The energy band gap (Eg) is obtained from the linear extrapolation up to the energy axis. The valence band top position was obtained from the VB XPS plot, and the conduction band (CB) bottom was calculated by subtracting the band gap value from VB position. The probable band structure was portrayed in potential vs NHE energy diagram.

Table S4. Calculation of VB and CB positions in potential vs NHE energy scale

Parameters	White (WZ)	Black (BZ)
Band gap (eV)	5.09	1.52
VB position	4.11	1.24
CB position	4.11-5.09= - 0.98	1.24-1.52= -0.28

- ✓ VB top position increment of BZ= 2.87 eV
- ✓ CB bottom position decrement of BZ = 0.7 eV

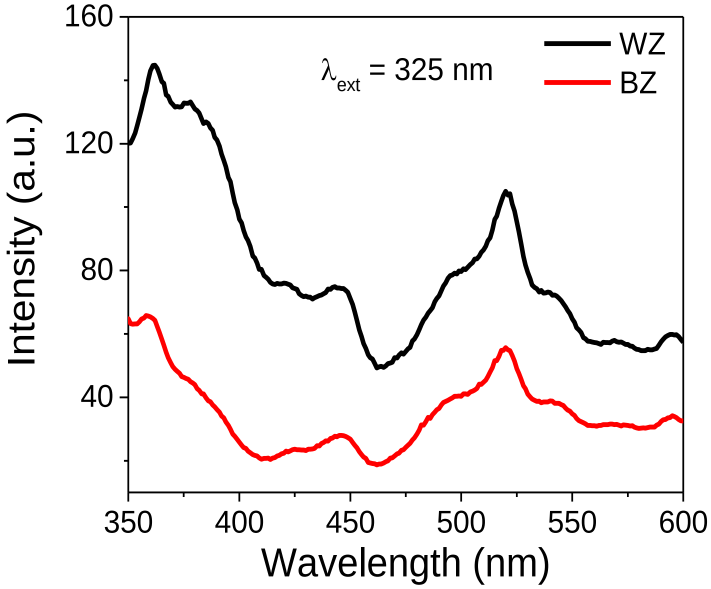

Figure S10. Photoluminescence spectra obtained from the solid film of white and black zirconia samples on glass plate using the same excitation wavelength (325 nm) and slit (5 nm)

- **Photocatalytic degradation of RhB in simulated sunlight**

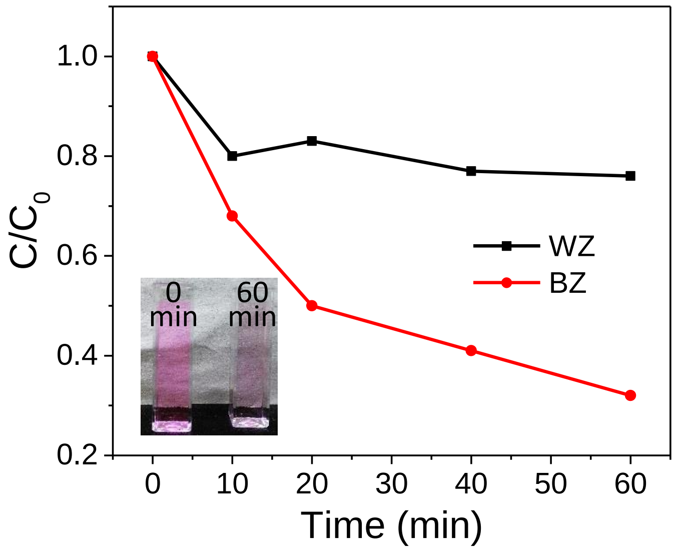

Figure S11. Degradation of Rhodamine B over WZ and BZ in the presence of solar light (1 sun) obtained from a solar simulator embedded with xenon light source and AM 1.5G filter. C_0 is the concentration RhB solution at the initial and C is the concentration of the solution after light irradiation determined from the absorbance of the solution. The inset figure is the photograph of the solution of RhB before and after 60 min light irradiation.

The enhanced solar light absorption of the black zirconia sample was further characterized by photocatalytic degradation of RhB. The result clearly indicates that WZ has almost no degradation ability in the presence of solar light, whereas the BZ shows sufficient degradation of RhB. The solar light-assisted photocatalytic performance of the BZ sample can be attributed to the improved absorbance in solar light, whereas WZ has almost no absorbance in solar light. This result further confirms the huge improvement of optical properties in our black ZrO_{2-x} compared with white ZrO_2. The initial decay in concentration in WZ may be refer to the normal absorption of RhB in the materials.

➢ Solar light-assisted hydrogen generation

Solar to hydrogen (STH) efficiency calculation:

We have calculated STH using the fallowing equations[1]

$$STH = \frac{Energy\ generate\ in\ the\ form\ of\ H_2\ (E_{H_2})}{Energy\ provided\ using\ AM1.5G\ solar\ simulator\ (E_{solar})} \times 100\%$$

$$E_{H_2} = Number\ of\ H_2\ molecules\ (N_{H_2}) \times Free\ energy\ of\ water\ splitting (\Delta G_w)$$

$$\Delta G_w = 2.46\ eV = (2.46 \times 1.602 \times 10^{-19})\ J = 3.941 \times 10^{-19}\ J$$

$$E_{solar} = Iluminated\ area \times incident\ power \times time$$

In the present work, the reaction was carried out in the presence of simulated sunlight (1sun, AM1.5G) obtained from a Newport solar simulator. The incident power is measured by a power meter and set at 1 sun (0.1 W) by changing the distance. 50 mg of catalyst (1% of Pt) was well dispersed in 50 ml 10 % methanol-water and studied. The approximate illuminated area (considering the cross-section of the cylindrical reactor that contains the solution) is 15.75 cm^2. So after 1 h, $E_{solar} = 5670\ J$. The rate of hydrogen generation for BZ is 0.505 $mmolg^{-1}h^{-1}$. Therefore, $E_{H_2} = 6\ J$ and $STH = \frac{E_{H_2}}{E_{solar}} * 100\% = 0.11\%$.

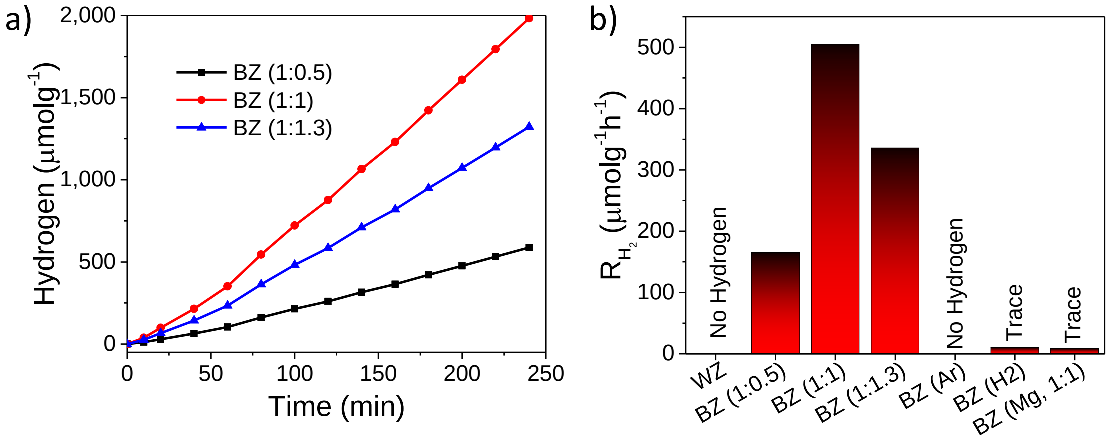

Figure S12. (a) Hydrogen generation profile obtained using different 1% Pt/BZ samples from 20% methanol-water solution under solar light (1 sun, AM 1.5G). (b) Rate of hydrogen (R_{H_2}) formation for different samples. The notation (x:x) is refers to the ratios of ZrO_2 and Mg, which are used for the sample preparation. The text in parentheses refers to specific condition like Ar: the sample prepared only in Ar atmosphere without Mg and H_2, H_2: the sample prepared in 5% H_2/Ar in the

absence of Mg, and Mg: the sample prepared with Mg in Ar atmosphere without H_2 for BZ (1:1) sample.

The BZ samples prepared with 1:05, 1:1, and 1:1.3 ratios exhibited a rate of hydrogen production of 165, 505, and 336 µmolg^{-1}h^{-1}, respectively, suggesting that the ratio (1:1) can be considered as an optimum ratio. Further, we have synthesized reduced zirconia using only hydrogen (5% H_2/Ar), only Mg (in Ar), and only Ar in the same reaction conditions to study the uniqueness of the present methods (see methods for details). All the materials were studied for the hydrogen production using the same experimental conditions. Interestingly, the material prepared using only Ar did not show any hydrogen production, whereas the materials prepared by only Mg and hydrogen show a little amount of hydrogen in the same experimental conditions under solar light. These results clearly demonstrate the requirement of both Mg and H_2 for the preparation of photocatalytically active black $ZrO_{2-x.}$

References

1 Liu, J. *et al.* Metal-free efficient photocatalyst for stable visible water splitting via a two-electron pathway. *Science* **347**, 970-974, (2015).
